# RETRACTION: Interleukin‐10‐Modified Adipose‐Derived Mesenchymal Stem Cells Prevent Hypertrophic Scar Formation via Regulating the Biological Characteristics of Fibroblasts and Inflammation

**DOI:** 10.1155/mi/9785923

**Published:** 2025-12-17

**Authors:** Mediators of Inflammation

RETRACTION: F. Xie, L. Teng, J. Lu, J. Xu, C. Zhang, L. Yang, X. Ma, and M. Zhao, “Interleukin‐10‐Modified Adipose‐Derived Mesenchymal Stem Cells Prevent Hypertrophic Scar Formation via Regulating the Biological Characteristics of Fibroblasts and Inflammation,” *Mediators of Inflammation*, no. 2022 (2022). https://doi.org/10.1155/2022/6368311.

The above article, published online on 21 June 2022 in Wiley Online Library (wileyonlinelibrary.com), has been retracted following the agreement of the journal’s Chief Editor Anshu Agrawal; and John Wiley & Sons Ltd.

The retraction has been agreed following an investigation of the concerns raised by *Hoya Camphorifolia* on PubPeer [1], which identified multiple instances of inappropriately overlapping figures as follows:

‐ In Figure 3a, the ADMSC and IL‐10 panels share overlapping features despite representing different experimental conditions. Additional concerns have been raised regarding the similarity of the Control panel to the WT‐Migration panel in Figure 5b of [2], where it represents a different cell type.
‐ The 5 day panel in Figure 5a appears identical to the CD3/Control panel shown in Figure 8e.

As a result, there are concerns regarding the reliability of the data presented and it is therefore retracted.

The authors have been informed of this retraction but did not provide a response.
